# Green Synthesis of Silver Nanoparticles Using Parthenium Hysterophorus: Optimization, Characterization and In Vitro Therapeutic Evaluation

**DOI:** 10.3390/molecules25153324

**Published:** 2020-07-22

**Authors:** Anam Ahsan, Muhammad Asim Farooq, Ali Ahsan Bajwa, Amna Parveen

**Affiliations:** 1 College of Animal Science & Veterinary Medicine, Shanxi Agricultural University, Taigu 030801, China; anamahsan267@gmail.com; 2 Department of Pharmaceutics, School of Pharmacy, China Pharmaceutical University, 211100 Nanjing, China; asim@stu.cpu.edu.cn; 3 Weeds Research Unit, New South Wales Department of Primary Industries, Wagga Wagga, NSW 2650, Australia; ali.bajwa@dpi.nsw.gov.au; 4 College of Pharmacy, Gachon University, Hambakmoero, Yeonsu-gu Incheon 406–799, Korea

**Keywords:** silver nanoparticles, parthenium hysterophorus, green chemistry, antimicrobial, anti-inflammatory, antioxidant, cytotoxicity

## Abstract

Traditional synthetic techniques for silver nanoparticles synthesis involve toxic chemicals that are harmful to humans as well as the environment. The green chemistry method for nanoparticle synthesis is rapid, eco-friendly, and less toxic as compared to the traditional methods. In the present research, we synthesized silver nanoparticles employing a green chemistry approach from *Parthenium hysterophorus* leaf extract. The optimized parthenium silver nanoparticles (PrSNPs) had a mean particle size of 187.87 ± 4.89 nm with a narrow size distribution of 0.226 ± 0.009 and surface charge −34 ± 3.12 mV, respectively. The physicochemical characterization of optimized SNPs was done by Fourier transform infrared spectroscopy (FTIR), thermogravimetric analysis (TGA) and differential scanning calorimetry (DSC). Moreover, the transmission electron microscopy (TEM) analysis indicates the spherical shape of NPs with an average diameter of 20–25 nm. PrSNPs were investigated for in vitro antibacterial, antifungal, anti-inflammatory, and antioxidant properties, and showed excellent profiles. The cytotoxic activity was analyzed against two cancer cell lines, i.e., B16F10 and HepG2 for 24 h and 48 h. PrSNPs proved to be an excellent anticancer agent. These PrSNPs were also employed for the treatment of wastewater by monitoring the *E. coli* count, and it turned out to be reduced by 58%; hence these NPs could be used for disinfecting water. Hence, we can propose that PrSNPs could be a suitable candidate as an antimicrobial, antioxidant, anti-inflammatory, and antitumor agent for the treatment of several ailments.

## 1. Introduction

Green chemistry is the utilization of chemistry principles to minimize the usage of poisonous chemicals, minimal unwanted residues, which might be injurious to human health as well as to the atmosphere [1,2]. A combination of green chemistry and nanotechnology is a versatile approach and has extended its consideration in the last few years [3]. The green synthesis of nanoparticle (NPs), which is ecological and cost-effective and employs stabilizing agents attained from plant extracts and other natural resources for the fabrication of NPs without the usage of harmful compounds, also promotes the sustainable use of NPs [4,5]. In the recent era, metallic NPs have been extensively used for various biomedical applications such as diagnosis, drug-delivery systems (DDs), and tissue engineering because of their exceptional physicochemical properties [6,7,8]. Among numerous metallic NPs, silver nanoparticles (AgNPs) have gained much attention for various applications, including imaging contrast agents, sensors, and antimicrobial agents owing to their better stability, electrical conductivity, and antimicrobial activity [9,10,11].

Also, AgNPs have shown their potential for the management of cancer. Many in vitro studies using AgNPs have proved their action as active anticancer agents against various cancer cells, including lung carcinoma, breast cancer, and human cervical cancer cells [12,13,14,15]. Generally, AgNPs are synthesized via several chemical and physical approaches. The physical methods consist of ball milling, flame sonication, radiation, electric arc discharge, pyrolysis, etc. [16,17]. These physical methods often require expensive instruments, higher pressure, and more energy consumption [18,19]. Though, the chemical approaches frequently need expensive metal salts and harmful solvents. Moreover, different stabilizers are also essential to avoid the aggregation of NPs to make them physiologically compatible [20,21,22]. In this context, green synthesis of AgNPs by plant extracts as a substitute method for the traditional physical and chemical NPs preparation methods [3,23].

*Parthenium hysterophorus* L. (Family-Asteraceae), generally recognized as parthenium weed or congress weed, is one of the most invasive plant species around the world [24]. Although conventional weed control methods are frequently used to manage this problematic plant, exploring its utility potential as an innovative management strategy is gaining attention nowadays [25]. The plant is well-known for its secondary metabolites, especially sesquiterpene, lactones, and phenolics that contribute towards its allelopathic capacity, cytotoxic activity, and allergenic reactions [25,26]. However, studies have reported that biochemicals from *Parthenium hysterophorus* also have medicinal values such as antioxidant, anti-inflammatory, antibacterial, antifungal, and anticancer activities [25,27,28,29]. There have been efforts in preparing NPs from the leaf extracts of *Parthenium hysterophorus,* and further work was suggested to develop the value-added products of biomedical significance [30]. Titanium dioxide nanoparticles have been synthesized using *P. hysterophorus* leaf extract through microwave irradiation method [31]. But no study is performed yet for the optimized synthesis of AgNPs from this plant and also detailed therapeutic investigation of the so-formulated NPs has been investigated.

The main objective of this study was the synthesis, optimization, and characterization of SNPs from *Parthenium hysterophorus,* as well as the detailed study of their therapeutic activity, which to our best knowledge has not been done so far. For attaining this purpose in this study, we fabricated silver NPs using *Parthenium hysterophorus* extract using a green chemistry approach. Synthesized PrSNPs were characterized by UV-Vis spectroscopy, and hydrodynamic size and surface charge on the NPs were measured by dynamic light scattering (DLS). The surface morphology of developed NPs was characterized by scanning electron microscopy (SEM) and transmission electron microscopy (TEM) microscopy, respectively. Moreover, the solid-state characterization had done by Fourier transform infrared spectroscopy (FTIR), thermogravimetric analysis (TGA), and differential scanning calorimetry (DSC). Besides, PrSNPs were investigated for in vitro antibacterial, antifungal, anti-inflammatory, and antioxidant properties, and showed excellent profiles. The cytotoxic effect of NPs was determined against two cancer cell lines, i.e., B16F10 and HepG2, for 24 h and 48 h.

## 2. Results and Discussion

### 2.1. Visual Inspection

**Figure 1:**
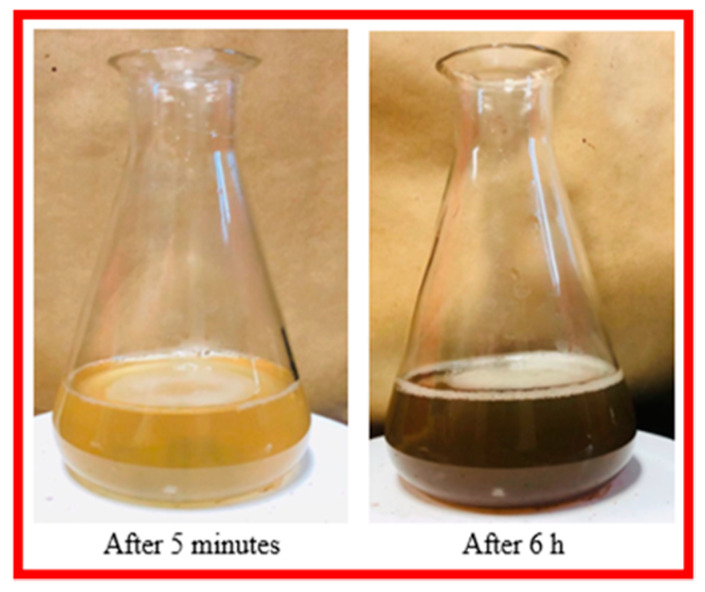
The color change of solution after the reduction of AgNO_3_ by leaf extract.

The change of color from light yellow to dark brown, thereby visually confirmed the production of silver nanoparticles. Color change was observed within a half-hour of exposure (Figure 1). The change in solution color is due to the surface plasmon resonance (SPR) displayed by the bioreduced AgNPs [9]. These results are in accordance with previously reported work [32,33].

### 2.2. Phytochemical Screening

The qualitative phytochemical screening of *P. hysterophorus* leaf extracts is given in Table S1. As presented, the extract consists of alkaloids, glycosides, proteins, phenols, terpenoids, flavonoids, saponins, and tannins. As previously studied, these phytochemicals are responsible for the reduction and capping of AgNPs [34,35,36].

### 2.3. UV-Visible Spectroscopy

**Figure 2:**
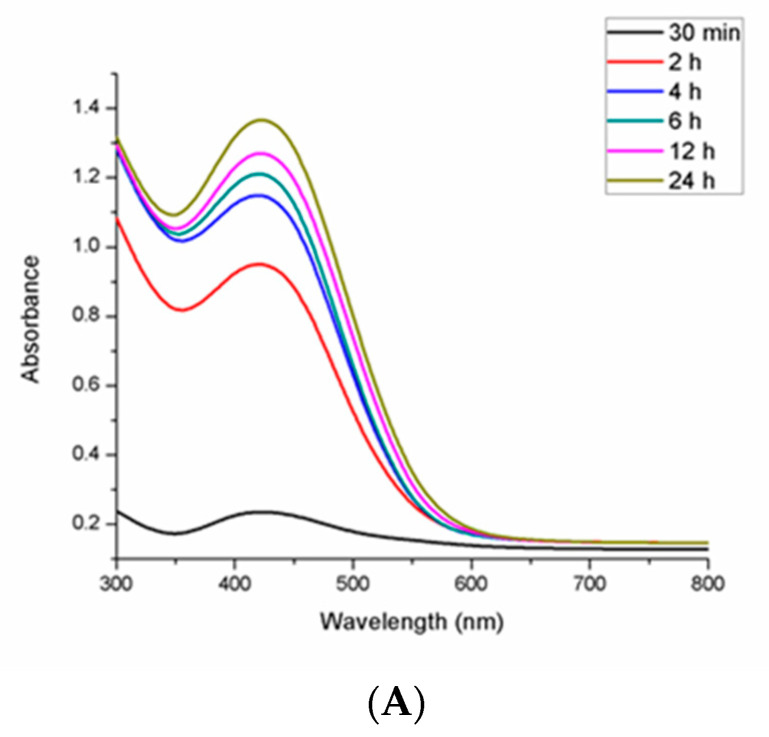

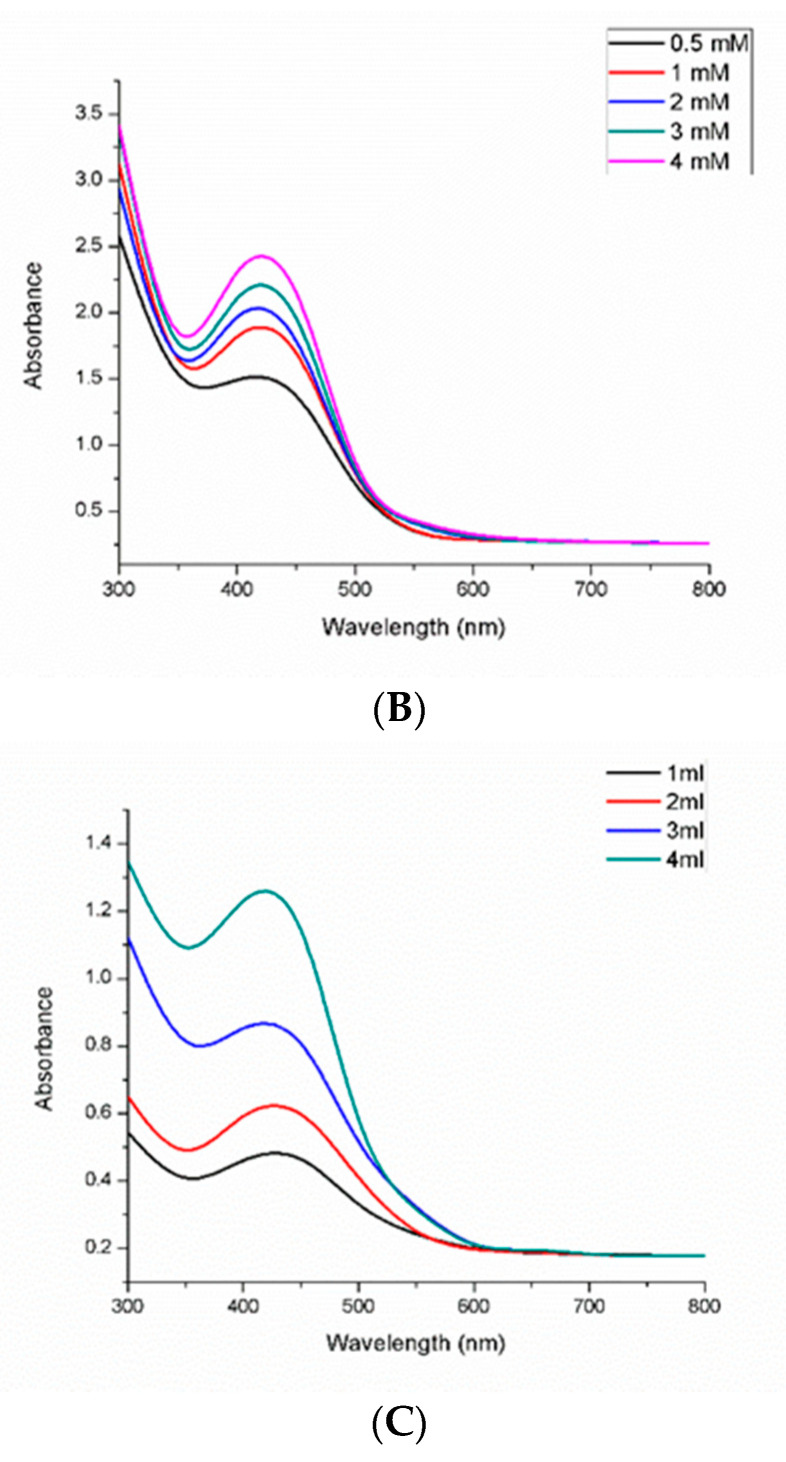
The UV-Visible spectrum of synthesized parthenium silver nanoparticles (PrSNPs) (**A**) as a function of time (**B**) as a function of silver nitrate concentration (**C**) and as a function of leaf extract concentration.

The ultraviolet-visible absorption spectrum of the synthesized PrSNPs dispersion was examined between 300–800 nm through a UV-visible spectrophotometer. The spectrum of the SNPs dispersion showed a single surface plasmon resonance absorption band with a maximum of approximately 432 nm (Figure 2). As the symmetry of NPs increased, the number of SPR peaks decreased, and spherical SNPs exhibited only one peak, while triangular and disc shape NPs showed two or more peaks. As the particle size increases, the SPR of the absorption spectrum of metal nanoparticles typically shifts to longer wavelengths [37].

The effect of varying concentrations of silver nitrate and leaf extract and reaction time on the formation of SNPs was also studied by periodically checking the absorbance of the reaction mixture through UV-Vis spectroscopy at 300–800 nm.

#### 2.3.1. Effect of Reaction Time

The optimal time for the synthesis of PrSNPs was examined through the UV-Vis spectra of the reaction mixture at various time intervals. As the reaction time increases, the redshift of the absorption peak designates an increase in the size of the NPs (Figure 2A). The increase in the intensity of the absorption peak over time is due to the increase in the number of absorbed metallic NPs [38]. The synthesis of SNPs from *Solanum trilobatum* extract was also studied by Vanaja et al., who observed a sharp narrow peak at 420 nm after 20 min of reaction indicating the synthesis of SNPs, which was shifted to 440 nm after 20 min. With an increase of reaction time, the SNPs production went on increasing, and the maximum absorption peak denoted the highest yield. The completion of the reaction occurred within 4 h, which was also visualized by precipitate formation at the bottom [39]. 

#### 2.3.2. Effect of Silver Nitrate Concentration

The optimal concentration for the synthesis of PrSNPs was found by varying concentrations of silver nitrate. The optimal concentration for NPs synthesis was found to be 1 mM (Figure 2B). Usually, by increasing the AgNO_3_ concentration, the fabrication of SNPs is increased, and the color of the solution is enhanced because the accumulation of silver ions takes place, and larger NPs are attained [40]. As the concentration of silver nitrate increases, the plasmon resonance (SPR) band becomes wider [39,41]. Similarly, 1mM silver nitrate was found to be optimal for the synthesis of silver nanoparticles from *Garcinia mangostana* extract [42].

#### 2.3.3. Effect of Leaf Extract Concentration

The reaction mixture was investigated by checking the UV-vis absorbance spectra at a varying concentration of the *P. hysterophorus* leaf extract. By increasing the concentration of leaf extract, the intensity of SPR band goes on increasing with maximum peak at the highest concentration of leaf extract (Figure 2C) which exhibited that by increasing the amount of plant extract, the chances of silver nitrate reduction increased leading to the development of stable and well-defined SNPs [43]. At an elevated concentration of the leaf extract, the biomolecules synergistically act as a reducing agent and as a surface coating of NPs and hence limit their aggregation [44]. Similar results were obtained when the silver nanoparticles were synthesized from *Malus domestica* [45], *Artemisia absinthium* [46], and *Pinus eldarica* [41].

### 2.4. DLS and Zeta Potential (Z.P) Analysis

**Figure 3:**
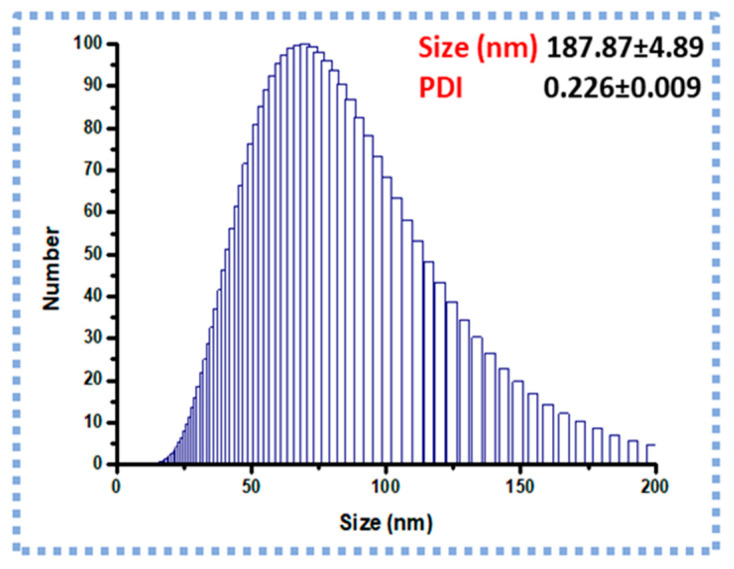
Particle size and size distribution of optimized nanoparticles (NPs) (*n* = 3).

The optimized PrSNPs had an average particle size of 187.87 ± 4.89 nm and Polydispersity index (PDI) 0.226 ± 0.009, respectively. As shown in Figure 3, the optimized NPs showed a narrow size distribution. Moreover, the Z.P of optimized NPs was found to be −34 ± 3.12 mV. The particle size measured by DLS is larger (187.87 ± 4.89 nm) than the particle size attained from the TEM images (20–25 nm) as the DLS measures the hydrodynamic diameter of the nanoparticles [47]. The PDI of PrSNPs dispersion was 0.226 ± 0.009, signifying a homogeneous and narrow particle size distribution [48,49].

### 2.5. SEM Investigation

**Figure 4:**
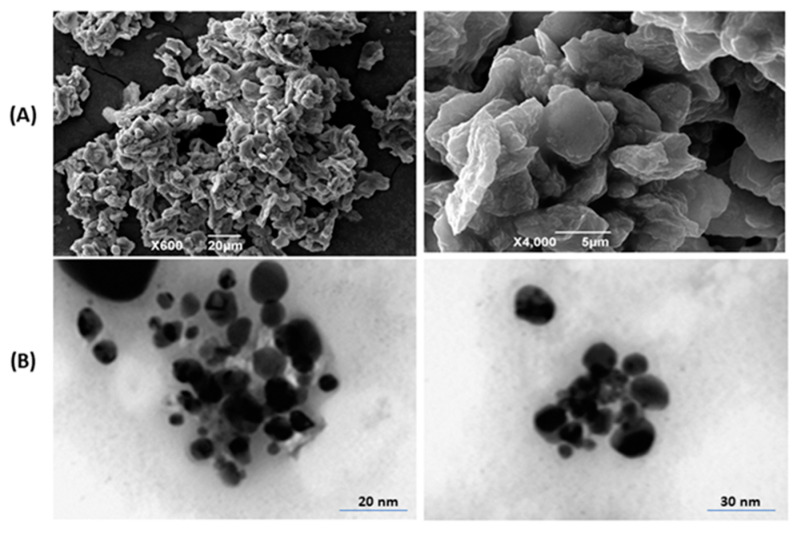
(**A**) Scanning electron microscopy (SEM) images of PrSNPs from different cross-sections at different magnification (×600, ×4000) and (**B**) transmission electron microscopy (TEM) images of optimized PrSNPs at different scale bars.

The SEM images of prepared PrSNPs showed that these nanoparticles are spherical. Some small NPs aggregated into larger particles, which is due to the existence of secondary metabolites occurring in the *P. hysterophorus* leaf extract (Figure 4A). The SEM figure showed a wide distribution of prepared NPs ranging from 10 to 130 nm in size.

### 2.6. TEM Analysis

The size and morphology of the synthesized PrSNPs were investigated by TEM. The majority of the nanoparticles exhibited uniform diameter and spherical shape (Figure 4B). The spherical shaped SNPs were having an average size of 20–25 nm. TEM results are following the SEM and DLS.

### 2.7. FTIR Analysis

**Figure 5:**
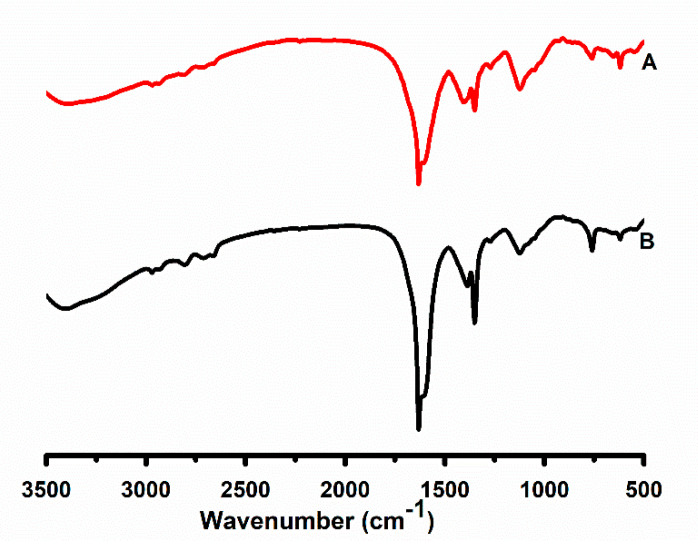
Fourier transform infrared (FTIR) spectra of *P. hysterphorus* leaf extract (**A**) and optimized PrSNPs (**B**).

FTIR characterized the leaf extract of *P. hysterophorus* and the resultant biogenic PrSNPs. The FTIR spectra for the leaf extract and PrSNPs are shown in (Figure 5A,B, respectively). The characteristic absorbance bands for *P. hysterophorus* and PrSNPs can be seen at 1631cm^−1^, 1588 cm^−1^, 1384 cm^−1^, 1400 cm^−1^, 1126 cm^−1^, 759 cm^−1^ at the wavelength range of 400–4000cm^−1^ which proved the presence of major functional groups of plant extract in the prepared NPs confirming capping and reduction of PrSNPs with the plant extract. 

An intense wide band at 3340 cm^−1^ could be associated with O–H, and N–H stretching is due to the presence of amide and amine of proteins found in the *P. hysterophorus* extract. These absorbance bands could be associated with stretching vibrations. A band of 1126 cm^−1^ in a specific area is most likely to occur from the C–O group polyol, i.e., catechins and hydroxy flavones. The complete vanishing of the band after biological reduction might be because polyols are primarily accountable for the reduction of silver ions, so they are oxidized as unsaturated carbonyl substrates, resulting in significant peaks at 1631 cm^−1^ to reduce silver ions. The detected peaks are primarily accredited to terpenoids and flavonoids, mostly present in the plant extract [50].

### 2.8. TGA and DSC Studies

**Figure 6:**
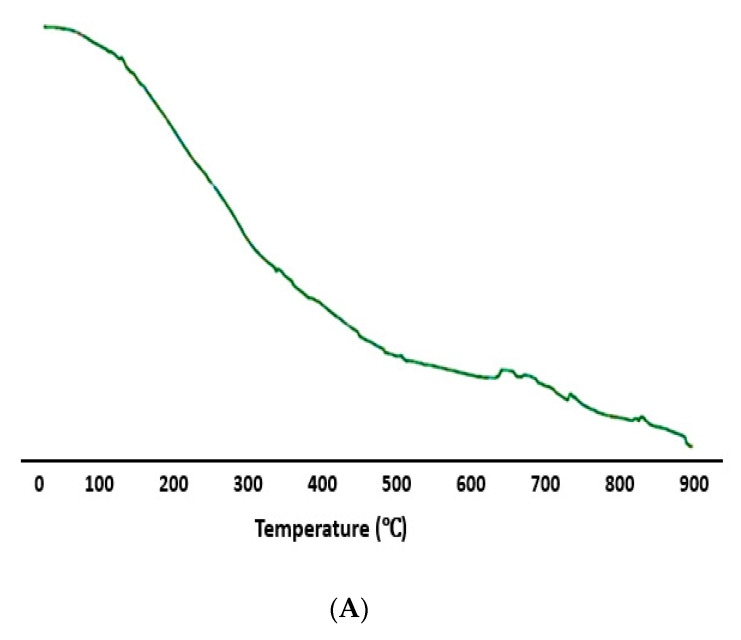

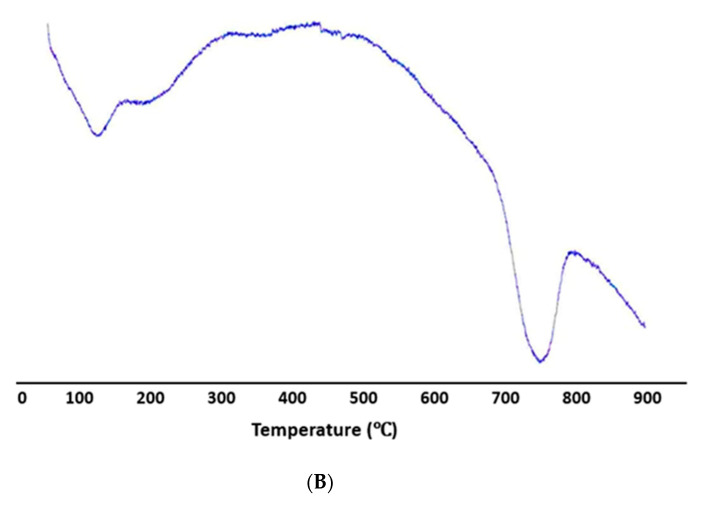
(**A**) Thermogravimetric analysis (TGA) and (**B**) differential scanning calorimetry (DSC) graphs of optimized PrSNPs.

Thermal studies were performed to investigate the thermal behavior of synthesized PrSNPs. TGA and DSC thermograms are shown in Figure 6A,B. It is evident from the TGA curve that the first weight loss of PrSNPs occurred in the temperature region of 200–300 °C, which continues to lose until 900 °C. Until 200 °C, silver nanoparticles did not show any characteristic weight loss. This weight loss is usually due to water evaporation, and loss of organic ingredients originated from plant extract [51]. 

The DSC graph exhibited a sharp exothermic peak between 200–600 °C, which is primarily due to the crystallization of PrSNPs, along with a small endothermic peak at 750 °C, which is due to metallic silver’s melting point [52]. 

The DSC curve showed that the PrSNPs had completely thermally decomposed and crystallized at the same time (Figure 6B). Both TGA and DSC analysis showed that PrSNPs were stable at temperatures up to 200 °C.

### 2.9. Antimicrobial Activity of Biogenic PrSNPs

For the determination of the antimicrobial activity of PrSNPs, bacterial, and fungal strains were tested by measuring their Minimum inhibitory concentration (MIC) and Minimum biocidal concentration (MBC) employing the microdilution broth method.

#### 2.9.1. Determination of MIC and MBC for Microbial Growth

The biosynthesized PrSNPs exhibited the highest MIC against *E. coli,* i.e., 28 μg/mL, while MBC against *E. coli* was twice as of MIC (38 μg/mL). The minimum MIC and MBC were recorded against *A. niger,* i.e., 57 μg/mL and 66 μg/mL, respectively. The MIC values against *S. aureus, P. aeruginosa, B. subtilis,* and *C. Albicans* was recorded as 31 μg/mL, 37 μg/mL, 43 μg/mL, and 49 μg/mL respectively. In contrast, MBC values were 37 μg/mL, 48 μg/mL, 45 μg/mL and 56 μg/mL respectively (Table S2). 

The MIC and MBC values for the *P. hysterophorus* leaf extract and standard drug against bacterial and fungal strains are also given in Table S2. The MIC and MBC values for the plant extract were high, showing less antimicrobial activity. It can be observed from (Table S2) that the MIC and MBC values of PrSNPs against *Escherichia coli, Bacillus subtilis, and Candida albicans* are quite comparable to the standard drugs which showed that our synthesized SNPs could be a suitable candidate for antibacterial and antifungal treatments. Our results in this study are better than the previously reported studies [35,53].

Silver nanoparticles have been reported to demonstrate good antimicrobial activity against different pathogens. They have been proven in numerous experiments, yet MIC values of the experiments vary depending on the materials used. Therefore, it is difficult to compare different results because there is no standard protocol for assessing the antimicrobial activity of NPs, and different researchers use different protocols [54]. Senthil et al. assessed the mechanism of action (MOA) of silver and observed cell damage by SEM. The bacterial cell wall is negatively charged, thus attracts silver ions. As a result, silver ions destroy cell membranes through protein denaturation and consequently cause cell death. This mechanism is also associated with the nature of the reaction of silver with phosphorus and sulfur compounds in biomolecules [55,56,57]. Compared with silver nitrate, silver nanoparticles have the less bactericidal capability, as silver salts not only cause cell damage but also cause DNA agglomeration [58].

#### 2.9.2. Anti-Inflammatory Activity of PrSNPs

Denaturation of protein is a renowned reason for inflammation. Therefore, in order to study the role of PrSNPs as an anti-inflammatory agent and also to investigate the mechanism that inhibits denaturation of protein, BSA was reacted with PrSNPs and diclofenac sodium to obtain comparative evidence on their effects [59].

**Figure 7:**
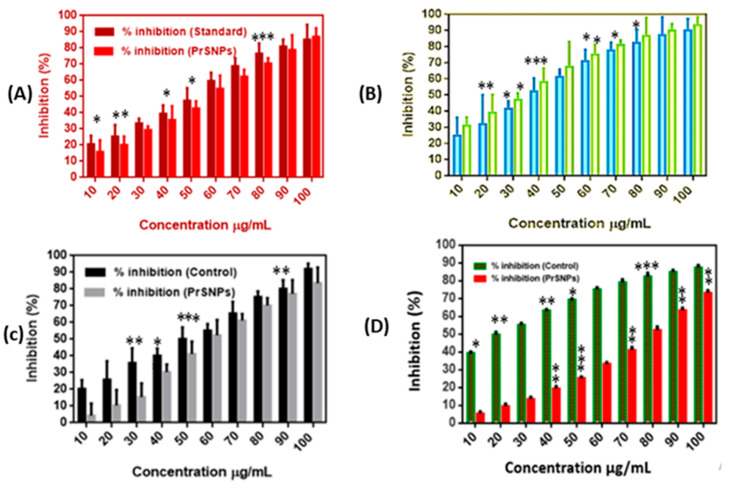
Anti-inflammatory activity of PrSNPs: (**A**) antioxidant activity of PrSNPs by DPPH assay (**B**) H_2_O_2_ assay (**C**) NO free radical and (**D**) nitric oxide radical scavenging assay (*n* = 3). All experiments performed in triplicate. * *p* < 0.05, ** *p* < 0.01 and *** *p* < 0.001, unpaired Student’s *t*-test.

The anti-inflammatory effect of PrSNPs exhibited a significant inhibitory effect on BSA protein denaturation (Figure 7A) As the concentration of SNPs increased, the level of inhibition increased. In the comparison of the standard drug (diclofenac sodium (628.5 µg/mL)), it has a significant anti-inflammatory effect. Therefore, it is evident that, in comparison to commercially available anti-inflammatory agents, PrSNPs exhibited significant inhibition of BSA protein denaturation even at lower concentrations. The synergistic anti-inflammatory effects of phytochemicals present in *P. hysterophorus* and PrSNPs can prove this. These results are in accordance with the previously reported literature [53,60,61].

It turns out that protein denaturation is among the many causes of inflammation. Protein denaturation leads to autoantigens production, which rarely causes inflammation in rheumatic diseases [62]. Previous studies have shown the role of anti-inflammatory drugs in the inhibition of protein denaturation, and also the mechanism of NSAID (Nonsteroidal anti-inflammatory drugs) drugs for inhibition of the denaturation of proteins at elevated temperatures and their use as commercial anti-inflammatory agents [63]. Therefore, an agent possessing protein denaturation inhibition activity could also be utilized for developing anti-inflammatory drugs. Also, marketed anti-inflammatory drugs may cause side effects such as stomach irritation. However, since the agent investigated in this research is of natural origin, it is anticipated to be harmless for the human body.

### 2.10. In Vitro Antioxidant Assay

To examine in vitro antioxidant property of PrSNPs, DPPH and H_2_O_2_ and NO radical scavenging assay was performed.

#### 2.10.1. DPPH Assay

PrSNPs have excellent DPPH scavenging activity at concentrations of 10–100 µg/mL, ranging from 31.17–93.45%, which is quite better than other similar studies. PrSNPs DPPH activity is also higher than control, i.e., Butylated hydroxytoluene (BHT) (Figure 7B). It shows that our green synthesized nanoparticles have excellent antioxidant properties. For example, a study reported DPPH scavenging potential of green synthesized silver nanoparticles at a concentration of 25 to 100 µg/mL ranging from 32-88%. Similarly, AgNPs synthesized by Colanitida’s bean valve extract exhibited excellent scavenging activity of DPPH at a gross margin of 32.81–100% at a concentration of 20 to 100 µg/mL [64]. So, we can conclude that our results are comparable to similar reported work.

#### 2.10.2. Hydrogen Peroxide (H_2_O_2_) Scavenging Assay 

The PrSNPs exhibited good H_2_O_2_ scavenging activity as 4.25–83.57% at a concentration of 10–100 µg/mL (Figure 7C), which is comparable to previously reported work. H_2_O_2_ scavenging activity of silver nanoparticles synthesized by *Helicteresisora* root extract was found to be 93.31%, while, for four different types of green synthesized silver nanoparticles, it was recorded as 77–99.8% [65,66]. The H_2_O_2_ scavenging ability of silver nanoparticles is essential for H_2_O_2_ degradation by catalysis becoming a key agent in self-care products, i.e., disinfectant or bleaching agents and broadly supplied in water or wastewaters.

It is also employed for paper and pulp bleaching, so it is an essential part of papermaking and recycling industrial wastewater [67]. Human exposure to H_2_O_2_ may lead to the production of extremely active hydroxyl (OH^_^) that enhances cell damage. Hydroxyl (OH^_^) has a vital role in inflammation, intestinal disease, aging, Alzheimer’s disease, and cancer. As a result, nanoplatforms can be used as an absolute means of removing H_2_O_2_ from environmental compartments, i.e., treating wastewater from the home and industrial resources. Silver nanoparticles could be analyzed for the detection of H_2_O_2_ [68].

#### 2.10.3. Nitric Oxide (NO) Radical Scavenging Assay

Nitric oxide is a significant biomodulated molecule in the neural, cardiovascular, and immune systems [69]. Biosynthetic PrSNPs exhibited a dose-dependent NO scavenging activity ranging from 5.71–73.41% at a concentration of 10–100 µg/mL (Figure 7D), and it was below the BHT (standard), i.e., 39.45–87.83% at the same concentration. The interaction among NO and PrSNPs at room temperature under anaerobic, dehydrated conditions facilitates the easy acceptance of electrons from AgNPs [63]. Biosynthetic silver nanoparticles from *C. tomentosum* leaf extract showed dose-dependency in reduction activity. The increase in AgNPs concentration continued to increase the reducing activity. PrSNPs have exhibited approximately equal reducing activity (73.41%) as of standard (BHT) i.e., 87.83% (Figure 7D). The reduction activity was due to the existence of phytochemicals in the extract [70]. These results are related to the results of other studies [71,72].

### 2.11. In Vitro Cytotoxicity Assay 

**Figure 8:**
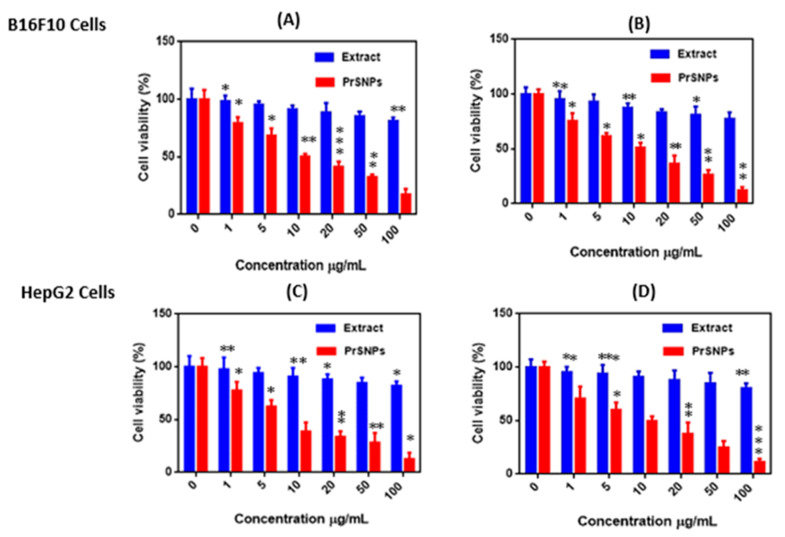
Cytotoxicity assay of PrSNPs against B16F10 cell line after treatment at 24 h (**A**) and 48 h (**B**) and HepG2 cell lines after treatment of 24 h (**C**) and 48 h (**D**) (*n* = 3). All experiments performed in triplicate. * *p* < 0.05, ** *p* < 0.01 and *** *p* < 0.001, unpaired Student’s *t*-test.

After 24 h and 48 h of treatment with varying concentrations of *P. hysterophorus* leaf extract and PrSNPs, B16F10 cells and HepG2 cells were used to evaluate the in vitro cytotoxicity assay. The results exhibited dose-dependent cytotoxicity (Figure 8). Cells not treated with PrSNPs were labeled as controls showing 100% cell viability. 

It is clear from the results that lower concentrations of leaf extract (1µg/mL and 5 µg/mL) were not toxic because the cell viability of both doses against both cell lines exceeded 90%. While at higher concentrations, leaf extract showed good cytotoxicity profile so we could predict that *P. hysterophorus* has antitumor activity. For PrSNPs, the cell viability has been dramatically reduced to < 70% against B16F10 cells while it is < 65% against HepG2 cells after 24h even at lower concentrations (5 µg/mL), which signifies the good cytotoxicity profile of PrSNPs. At higher concentrations of PrSNPs, the cytotoxicity increased significantly as the cell viability dropped to about 12.54% against HepG2 cells while 17.24% against B16F10 cells after 24 h. The IC_50_ values for developed nanoparticles against both cell lines (B16F10 cells& HepG2 cells) were 10.18 ± 0.79 µg/mL, 10.531 ± 1.15 µg/mL and 8.016 ± 1.12 µg/mL, 9.87 ± 2.45 µg/mL for 24 and 48 h, respectively.

The cell viability further decreased in a dose-dependent manner against both cell lines after 48 h of incubation. These cytotoxic results of PrSNPs are better as compared to previously reported work [73,74,75,76]. So, we can conclude that PrSNPs could prove to be a potential candidate for cancer treatment after further investigation.

### 2.12. Treatment of Wastewater by PrSNPs

The untreated wastewater demonstrated that *E. coli* contamination was about 50 CFU/mL. After an hour of treatment, the amount of CFU/mL began to decrease to 58% of the initial bacterial count. This behavior signifies that PrSNPs can be employed as a supplementary method for disinfecting wastewater. Lovatel et al. immersed silver nanoparticles in a mixture of montmorillonite and alginate, and after 90 min of wastewater disinfection, the bacterial concentration decreased by 98.5% [77]. So, we can say that PrSNPs have sufficient antibacterial potential to treat wastewater, particularly those contaminated with bacteria, i.e., *E. coli.*

## 3. Materials and Methods

### 3.1. Materials

Silver nitrate, Bovine Serum Albumin (BSA), and other chemicals were purchased from Sigma Aldrich (Auburn, AL, USA). Murine skin melanoma cell line B16F10 cells and human hepatocellular carcinoma cell line HepG2 were purchased from the Chinese Academy of Sciences Cell Bank, Shanghai, China. RPMI-1640, Dulbecco’s modified Eagle’s Medium (DMEM), Fetal bovine serum (FBS), and trypsin, and phytochemical screening reagents were bought from Thermo Fisher Scientific, Inc. (Waltham, MA, USA). All other ingredients, solvents, and reagents were of analytical grade. Double-distilled water was employed in all the experiments.

### 3.2. Methods

#### 3.2.1. Preparation of Leaf Extract

**Figure 9:**
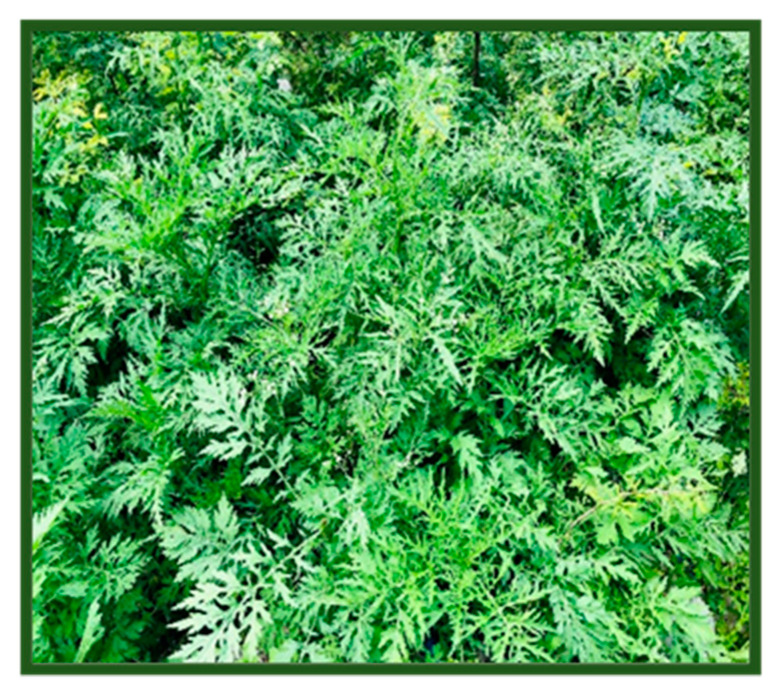
Parthenium hysterophorus plant.

Fresh green leaves of *Parthenium hysterophorus* (Figure 9) were collected from Chenab Nagar, Pakistan. Leaves were thoroughly rinsed with tap water and then with double-distilled water. The leaves were shade dried for two weeks. Uniform powder was made by passing the dry leaves through a 50 mm sieve. To prepare leaf extract, 10 g of leaf powder was mixed with 100 mL distilled water followed by boiling at 80 °C for 30 min. Then, the extract was filtered by Whatman filter paper (No. 1) and stored at 4 °C before being used to biosynthesize silver nanoparticles.

#### 3.2.2. Phytochemical Analysis

*Parthenium hysterophorus* leaf extract was passed through phytochemical screening [78].

##### Mayer’s Test (Alkaloids)

Two to three droplets of Mayer’s reagent were mixed with 1mL of leaf extract. The formation of pale yellow or white precipitate shows alkaloid presence.

##### Molisch’s Test (Carbohydrates and Glycosides)

A volume of 300 μL of Molisch’s reagent along with three drops of H_2_SO_4_ was mixed with 1 mL leaf extract. Reddish color formation indicates the presence of carbohydrates and glycosides.

##### Biuret Test (Proteins)

Two milliliters of protein solution was added in 1 mL of leaf extract. Then 2–3 drops of alkali were added and thoroughly mixed. Then dropwise we added copper reagent with constant vertexing till the appearance of purple color.

##### Ninhydrin Test (Amino acid)

A volume of 300 μL of Ninhydrin reagent and 1 mL of distilled water was added to 1 mL of leaf extract followed by boiling for ten minutes. The appearance of dark purple color shows protein presence.

##### Ferric Chloride Test (Phenols)

One milliliter of leaf extract was mixed with 2 mL of ferric chloride. The appearance of the reddish-brown color indicates phenol’s presence.

##### Neutral Ferric Chloride Test (Tannins)

Two milliliters of 5% ferric chloride were mixed with 1 mL of leaf extract. The appearance of greenish-black/dark blue color indicates the presence of tannins.

##### Shinoda Test (Flavonoids)

One milliliter of 10% lead acetate solution was added to 1 mL of leaf extract. The formation of yellow color precipitates confirms the presence of flavonoids.

##### Salkowski’s Test (Terpenoids)

A volume of 300 μL of leaf extract was added to 2 mL of chloroform, and then 3 mL concentrated H_2_SO_4_ was added to the layer. The appearance of a reddish-brown color shows terpenoids’ presence.

##### Froth Test (Saponins)

Leaf extract was mixed with 2 mL of distilled water and briskly shaken. Stable broth formation shows saponin’s presence.

### 3.3. Phytoreduction of Parthenium Silver Nanoparticles (PrSNPs)

For silver nanoparticles synthesis, 1 mL of the *P. hysterophorus* leaf extract was added dropwise into 9 mL of freshly prepared 1 mM AgNO_3_ solution with continuous stirring at room temperature for 30 min. The synthesis of nanoparticles was confirmed by reddish-brown color. After that solution was kept in the dark for a further 24 h allowing the complete reduction of the silver nitrate solution. UV-Vis spectrophotometry also confirmed the synthesis of silver nanoparticles. Lastly, PrSNPs were collected by centrifugation at 10,000× *g* rpm for 15 min by using optimized conditions [79].

Different concentrations of AgNO_3_ i.e., 0.5 mM, 1 mM, 2 mM, 3 mM, and 4 mM and leaf extract i.e., 1 mL, 2 mL, 3 mL, and 4 mL and different reaction time i.e., 30 min, 1 h, 4 h, 6 h, 12 h, and 24 h were employed to standardize the optimum conditions.

### 3.4. Visual Inspection

The color change after mixing silver nitrate with the plant extract in a suitable ratio is the first indicator of the formation of silver nanoparticles [9,80,81]. The color change was monitored at various time intervals after reducing silver nitrate with *P. hysterophorus* leaf extract for checking the synthesis of PrSNPs.

### 3.5. UV-Visible Spectroscopy

The synthesis of silver nanoparticles was investigated by checking the absorbance of the diluted solution of silver nanoparticles suspension at a wavelength of 300–800 nm by a UV-Vis spectrophotometer (Thermo Fisher Scientific, Austin, TX, USA). Silver nanoparticles exhibit strong electromagnetic wave absorption in the visible range owing to the surface plasmon resonance (SPR).

### 3.6. Dynamic Light Scattering (DLS) and Zeta Potential (Z.P) Analysis

The average hydrodynamic diameter, size distribution, and polydispersity index (PDI) of NPs was measured by using a Particle Size Analyzer (Brookhaven, NY, USA) at 25 °C and a scattering angle of 90°. Briefly, 3 mg of PrSNPs were dispersed in 3 mL of double distilled water following sonication for 5 min. For surface charge measurement of developed NPs, Zeta Pals (Brookhaven, NY, USA) was utilized.

### 3.7. Scanning Electron Microscopy (SEM)

Morphology of the synthesized PrSNPs was investigated by scanning electron microscope (JSM-5600, Tokyo, Japan). For analysis, lyophilized PrSNPs were placed on glass slide following coating them with a thin layer of gold-palladium blend once mounted them on a carbon grid.

### 3.8. Transmission Electron Microscopy (TEM)

The size and shape of silver nanoparticles were investigated by TEM. A drop of biosynthetic SNPs dispersion, i.e., 1 µg/mL, was kept on carbon-coated copper mesh following drying at room temperature. Transmission electron microscope (JEOL TEM, Tokyo, Japan) captured photomicrographs of the samples.

### 3.9. Fourier Transform Infrared Spectroscopy (FTIR)

FTIR analysis was used to scan the synthesized silver nanoparticles to obtain the capped functional groups on the nanoparticles. FTIR analysis of the *P. hysterophorus* leaf extract and synthesized nanoparticles was carried out using the KBr pellet method by the FTIR apparatus (Bruker, Auburn, AL, USA), in transmittance mode from 400–4000 cm^−1^ at 4 cm^−1^.

### 3.10. Thermogravimetric Analysis (TGA) and Differential Scanning Calorimetry (DSC)

The thermal stability of synthesized Ag nanoparticles was evaluated by performing TGA and DSC characterization by Hitachi thermal analysis system (Hitachi, Austin, TX, USA). TGA was used to study the weight loss of surface-coated SNPs. A standard differential scanning calorimeter investigated the thermal behavior of PrSNPs. PrSNPs obtained after lyophilization was employed for TGA and DSC analysis. We washed the alumina crucible with acetone and tied to another crucible (reference), keeping them close while avoiding the direct contact. 9.3 mg of PrSNPs was placed in the crucible, heating from 40–900 °C at the rate of 10 °C min^−1^, and a constant nitrogen flow rate of 40 mL/min was maintained. The nitrogen level was periodically checked during analysis.

### 3.11. Antimicrobial Activity of Biogenic PrSNPs

The antimicrobial activity of the silver nanoparticles was evaluated using a microdilution broth method by determining their MIC and MBC. MIC is known to be the minimum concentration of SNPs, where no visible fungal or bacterial growth occurs [82]. The biosynthesized AgNPs were evaluated against Gram-positive bacteria (*B. subtilis* and *S. aureus*), Gram-negative bacteria (*P. aeruginosa,* and *E. coli*) and fungi (*A. niger and C. Albicans*). Ampicillin (antibacterial) and fluconazole (antifungal) were used as standard drugs.

#### Determination of Minimum Inhibitory Concentration (MIC) and Minimum Biocidal Concentration (MBC) for Microbial Growth

Measurements were performed using triplicate 96-well microtiter plates. Nutrient broth culture medium (NB) was used for bacterial growth, while for fungal growth Sabouraud’s dextrose broth (SBD) was used. A final concentration of 4 × 10^5^ c.f.u. of bacteria or fungi at mL^−1^ was preserved in each well of the microtiter plate. PrSNPs were prepared at different concentrations (10–100 µg/mL).

Microbial inoculum mixed broth (positive control) and non-inoculated sterile broth (negative control) were also preserved. Microbial inoculum density was assessed from colony counts. The inoculated microtiter plates were incubated for 24 h for bacteria or 48 h for fungi at 37 °C. Then visually checked the MIC values. The minimum biocidal (fungicidal or bactericidal) concentration (MBC) of PrSNP against microorganisms has also been determined, which refers to the lowermost concentration of the antimicrobial agent, which can prevent 99.9% of microbial cell growth. Following incubation, each test sample (100 µL) was spread on antimicrobial-free medium (NB for bacteria and SDA for fungi). The plates were incubated for 24 or 48 h at 37 °C, and the growth of microorganisms was observed.

### 3.12. In Vitro Anti-Inflammatory Assay

Different concentrations (10–100 µg/mL) of PrSNPs were added to 1% aq. solution of bovine serum albumin and the mixture’s pH was set at 6.5 by adding few drops of 1N HCl. The samples were then incubated for 20 min at 37 °C following heating at 60 °C for 20 min in a water bath. When the sample was cooled, absorbance at 660 nm was recorded by the spectrophotometer. Diclofenac sodium was considered a standard [83]. All experiments were performed in triplicate manner. The percentage inhibition of denaturation of protein was determined by Equation (1).

(1) $$\%\;\mathrm{inhibition}=\frac{{\mathrm{Abs}}_{\mathrm{control}}-{\mathrm{Abs}}_{\mathrm{smaple}}}{{\mathrm{Abs}}_{\mathrm{control}}}\times 100\%,$$

### 3.13. In Vitro Antioxidant Assay

For evaluating in vitro, the antioxidant activity of the synthesized PrSNPs, H_2_O_2_, DPPH, and NO scavenging activity was analyzed.

#### 3.13.1. DPPH (2,2-diphenyl-2-picrylhydrazyl) Assay

The free radical scavenging activity of PrSNPs was measured by the DPPH method. A volume of 500 μL of DPPH solution (0.3 mM, 0.6 mg of DPPH dissolved in 12 mL of methanol) was added at varying concentrations (10–100 μg/mL) to 3 mL of PrSNPs aqueous solution. Controls were prepared by the addition of 3 mL of water to 500 μL DPPH. The measurement was repeated three times to obtain an average value. The anti-free-radical activity was calculated by determining the reduction in absorbance (after 30 min at 517 nm) after the test sample was added, expressed as the percent inhibition of DPPH free radicals (% PI) [84]. The scavenging ability of DPPH free radicals was determined using Equation (1).

#### 3.13.2. Hydrogen Peroxide (H_2_O_2_) Assay

A volume of 0.6 mL of H_2_O_2_ (5 mM) formulated in phosphate buffer at pH 7.4 was mixed with PrSNPs with concentration ranging from 10–100 µg/mL. The mixture was then held for 10–15 min at room temperature, and absorbance was recorded at 610 nm by UV-visible spectrophotometer [85]. Ascorbic acid was employed as a control. The % inhibition was determined by Equation (1).

#### 3.13.3. Nitric oxide (NO) Radical Scavenging Assay

Nitric oxide scavenging activity was evaluated by using modified methods to measure nitrite ions production by interacting sodium nitroprusside with oxygen in physiological pH by employing Griess reaction reagent. In short, nitric oxide radicals formed from 20 mM of sodium nitroprusside (100 µL), incubated with 100 µL PrSNPs (10–100 µg/mL) at room temperature for 60 min. NO, and BHT (Butylated hydroxytoluene) scavengers were employed as a positive control [85]. For determination of nitric oxide radical scavenging activity, Equation (1) was used.

### 3.14. In Vitro Cytotoxicity Assay

B16F10 and HepG2 cells were grown in a supplement containing 10% of FBS in the essential DMEM and RPMI-1640, respectively, and 1% antibiotic solution (amphotericin B and streptomycin) in a humid environment with a temperature of 37 °C and a humidity of 95% oxygen and 5% CO_2_. In short, cell lines were seeded at 3 × 10^4^/well in a 96-well microplate, performed in triplicate, and allowed to grow at 37 °C for 24 h. After 24 h of incubation, different concentrations of PrSNPs (0–100 μg/mL) were added to each well for 24 and 48 h. By the end of treatment, the medium was discarded, and incubated cells and 100 μL MTT (5 mg/mL in phosphate buffer saline at pH 7.4) was added to fresh medium in the dark at 37 °C for 4 h. After 4 h, mitochondria reduced MTT to form formazan crystals and was mixed in DMSO (150 μL/well) and placed on a microplate reader (Spectramax) and checked OD at 570 nm. Cells without any treatment were used as control throughout the experiment [86]. The following Equation (2) was used to determine cell viability:

Cell viability (%) = OD _Sample_ ×OD _Control_ × 100 (2)

### 3.15. Treatment of Water by PrSNPs

Wastewater samples (250 mL) were treated with PrSNPs (10–100 µg/mL) by shaking at 200 rpm for 30–100 min. Quantitative assessment of *E. coli* was performed using Petrifilm 3M^®^, according to AOAC official method 991.14, for the assessment of treated wastewater [87].

### 3.16. Statistical Analysis

All the experiments were done thrice, and data were expressed as mean ± standard deviation (SD) by one-way ANOVA and Graph Pad Prism version 5.

## 4. Conclusions

The green chemistry approach was employed for the rapid and effective synthesis of silver nanoparticles using *Parthenium hysteorphorus* leaf extract without any harmful side effects. These synthesized PrSNPs were successfully optimized by UV-Vis spectroscopy by using different ratios of silver nitrate and leaf extract, and reaction time. Phytochemical screening of the *P. hysterophorus* leaf extract was done to confirm which functional groups are present in the extract which was further confirmed by FTIR analysis when the detected function groups (Flavonoids, terpenoids, steroids, amides, alkaloids, and tannins) caused the reduction and stabilization of the synthesized NPs. The size and morphology of the PrSNPs were examined by DLS, SEM, and TEM, confirming the spherical-shaped nanoparticles with size ranging from 20–25 nm.

Further characterization was performed by DSC, TGA, and zeta potential measurements. The synthesized SNPs exhibited excellent antibacterial, antifungal, anti-inflammatory, antioxidant, and anticancer profiles in a dose-dependent manner. It confirms they are potential candidates as antimicrobial, anti-inflammatory, and antioxidant and anticancer agents. Their role as wastewater disinfectant was also analyzed by measuring the *E. coli* count in the wastewater after treatment with synthesized PrSNPs, and it showed to be decreased by 58%, confirming its role in disinfecting wastewater. 

## Supplementary Materials

The following are available online, Table S1: Phytochemical screening of *P. hysterophorus* leaf extract, Table S2: MIC and MBC values (μg/mL) of plant extract, PrSNPs and standard drugs against bacterial and fungal strains.
