# Expression of Concern: Ahsan et al. Green Synthesis of Silver Nanoparticles Using Parthenium Hysterophorus: Optimization, Characterization and In Vitro Therapeutic Evaluation. *Molecules* 2020, *25*, 3324

**DOI:** 10.3390/molecules31132219

**Published:** 2026-06-24

**Authors:** Molecules Editorial Office

**Affiliations:** MDPI, Grosspeteranlage 5, 4052 Basel, Switzerland; molecules@mdpi.com

The Editorial Office has been made aware of potential issues with the integrity of data presented in Figure 3 of this article [1]. Namely, there is a discrepancy between the size distribution and particle size reported. The authors have been contacted and requested to provide clarification as well as the original data.

At this stage, the investigation is ongoing, and no final conclusion has been reached. Therefore, the Editorial Office would like to alert readers that the reliability of the data presented in this article is currently under assessment. Readers are advised to interpret the results with caution.

Further editorial action will be taken, if necessary, once the investigation has been completed. The journal remains committed to maintaining high standards of publication ethics and will update this notice as appropriate.
