# Supplementary material for: Green Synthesis of Silver Nanoparticles Using Parthenium Hysterophorus: Optimization, Characterization and In Vitro Therapeutic Evaluation
Source: Molecules. 2020 Jul 22;25(15):3324. doi: 10.3390/molecules25153324 (PMC7435648; doi:10.3390/molecules25153324)
Supplement: Supplementary file 1 [file molecules-25-03324-s001.pdf]

Supplementary

Table S1. Phytochemical screening of *P. hysterophorus* leaf extract

Phytochemicals	Ethanol extract	Methanol extract
Alkaloids	+	+
Carbohydrates	+	+
Glycosides	+	+
Proteins	+	+
Amino acids	+	+
Phenols	+	+
Tannins	-	+
Flavonoids	+	+
Terpenoids	+	+
Saponins	+	-

Table S2. MIC and MBC values (µg/mL) of plant extract, PrSNPs and standard drugs against bacterial and fungal strains

**Microorganism**	**MIC**			**MBC**		
	**Plant extract**	**PrSNPs**	**Standard drug**	**Plant extract**	**PrSNPs**	**Standard drug**
Staphylococcus aureus	89	31	20	176	37	32
Bacillus subtilis	94	43	37	198	45	46
Escherichia coli	67	28	25	153	38	39
Pseudomonas aeruginosa	71	37	30	182	48	51
Candida albicans	101	49	38	201	56	64
Aspergillus niger	112	57	29	209	66	58
